# A Novel Phosphatase Inhibitor May Be a STEP Toward Ameliorating Cognitive Dysfunction

**DOI:** 10.1371/journal.pbio.1001924

**Published:** 2014-08-05

**Authors:** Richard Robinson

**Affiliations:** Freelance Science Writer, Sherborn, Massachusetts, United States of America

The ultimate cause of Alzheimer's disease (AD) may be accumulation of the excess proteins found in plaques and tangles in the brain, but the immediate causes of cognitive dysfunction in the disease likely lie several steps downstream from the disruption of protein homeostasis. One implicated pathway involves striatal-enriched protein tyrosine phosphatase, or STEP, a neuron-specific enzyme that, among other jobs, regulates the trafficking of synaptic glutamate receptors and the activity of a group of widely active kinases. STEP is overactive in AD, in part because it isn't degraded fast enough, and its overactivity disrupts the post-synaptic events that underlie learning and memory. In animal models of AD, knocking out STEP improves cognition. Thus, STEP inhibition is a potential target for treatment of AD. In this issue of *PLOS Biology*, Jian Xu, Paul Lombroso, and colleagues report their discovery of a new class of STEP inhibitor—a discovery that involved a small but significant bit of serendipity—and demonstrate its potential in an AD animal model.

**Figure 1:**
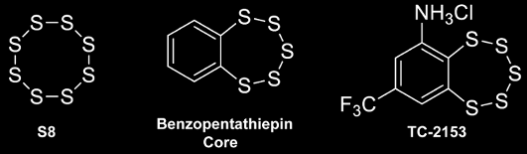
From impurity to drug. On the left is the normal cyclic eight-atom form of elemental sulfur, present as an impurity in some of the drug samples. In the center is the related compound benzopentathiepin, which contains five sulfur atoms attached in a loop to a benzene ring. On the right is a derivative of benzopentathein, TC-2153, which has more desirable pharmacological properties.

The authors began by conducting a high-throughput screen of 150,000 compounds, testing the ability of each to inhibit STEP's phosphatase activity. As is usual in such screens, a number of good candidates emerged. These were winnowed down to eight, chosen for their high activity at low concentration and favorable properties, such as likely ability to cross the blood-brain barrier and absence of known toxic moieties, all important for developing a centrally active drug. Following standard practice, next, they synthesized the molecules from scratch, and here got a surprise—the compounds displayed little STEP inhibitory activity. Some chemical detective work revealed the true inhibitor was elemental sulfur, S_8_, present as an impurity in the commercially obtained samples used in the screening. This ring compound doesn't make a good drug, so the authors investigated a structurally related compound, benzopentathiepin, containing a ring of six carbons fused to a ring of five sulfurs. A derivative, TC-2153, was known to have low toxicity and was likely to cross the blood-brain barrier, and, they found, was a potent inhibitor of STEP.

They showed that TC-2153 increased the phosphorylation state of multiple STEP substrates, both in cell culture and in mice. It was also relatively specific for STEP, with little inhibition of related phosphatases in noncortical neurons (STEP is restricted to the central nervous system), or in the periphery (where STEP is not expressed). Exactly how TC-2153 inhibits STEP is still under investigation, but it appears to interact with sulfurs in the enzyme's catalytic site, forming an irreversible covalent linkage with them.

To test whether TC-2153 could reverse some of the cognitive effects of STEP overactivity, the authors turned to the “triple transgenic” mouse model, with mutations in three genes known to cause AD: presenilin 1, amyloid precursor protein, and tau. Compared to vehicle, intraperitoneal injection of TC-2153 improved spatial working memory, novel object recognition, and reference memory, all standard tests of cognitive function in AD models. The treatment had no effect on either a-beta, found in amyloid plaques outside of cortical neurons, or phospho-tau, found in neurofibrillary tangles inside them, indicating that the beneficial effect of TC-2153 was not due to alteration of events upstream of STEP overactivity.

The fortuitous discovery of this novel class of protein tyrosine phosphatase inhibitors is likely to lead to further development of potential drugs for AD, as well as several other neuropsychiatric and neurodegenerative diseases in which STEP is overactive. Whether any of these can become effective treatments for these diseases is, of course, a far more challenging question, given the disappointing track record of drug discovery in this field to date. But the demonstration that cognitive dysfunction can be ameliorated through inhibition of this enzyme may lead to better understanding of how that dysfunction arises, which by itself is no small…step.

**Xu J, Chatterjee M, Baguley TD, Brouillette J, Kurup P, et al. (2014) Inhibitor of the Tyrosine Phosphatase STEP Reverses Cognitive Deficits in a Mouse Model of Alzheimer's Disease.** doi:10.1371/journal.pbio.1001923

